# Signaling of Pigment-Dispersing Factor (PDF) in the Madeira Cockroach *Rhyparobia maderae*

**DOI:** 10.1371/journal.pone.0108757

**Published:** 2014-09-30

**Authors:** Hongying Wei, Hanzey Yasar, Nico W. Funk, Maria Giese, El-Sayed Baz, Monika Stengl

**Affiliations:** University of Kassel, FB 10, Biology, Animal Physiology, Kassel, Germany; University of Texas Southwestern Medical Center, United States of America

## Abstract

The insect neuropeptide pigment-dispersing factor (PDF) is a functional ortholog of vasoactive intestinal polypeptide, the coupling factor of the mammalian circadian pacemaker. Despite of PDF's importance for synchronized circadian locomotor activity rhythms its signaling is not well understood. We studied PDF signaling in primary cell cultures of the accessory medulla, the circadian pacemaker of the Madeira cockroach. In Ca^2+^ imaging studies four types of PDF-responses were distinguished. In regularly bursting type 1 pacemakers PDF application resulted in dose-dependent long-lasting increases in Ca^2+^ baseline concentration and frequency of oscillating Ca^2+^ transients. Adenylyl cyclase antagonists prevented PDF-responses in type 1 cells, indicating that PDF signaled via elevation of intracellular cAMP levels. In contrast, in type 2 pacemakers PDF transiently raised intracellular Ca^2+^ levels even after blocking adenylyl cyclase activity. In patch clamp experiments the previously characterized types 1–4 could not be identified. Instead, PDF-responses were categorized according to ion channels affected. Application of PDF inhibited outward potassium or inward sodium currents, sometimes in the same neuron. In a comparison of Ca^2+^ imaging and patch clamp experiments we hypothesized that in type 1 cells PDF-dependent rises in cAMP concentrations block primarily outward K^+^ currents. Possibly, this PDF-dependent depolarization underlies PDF-dependent phase advances of pacemakers. Finally, we propose that PDF-dependent concomitant modulation of K^+^ and Na^+^ channels in coupled pacemakers causes ultradian membrane potential oscillations as prerequisite to efficient synchronization via resonance.

## Introduction

The accessory medulla (AMe), the circadian pacemaker of cockroaches and fruit flies [1], and the suprachiasmatic nucleus (SCN), the mammalian circadian clock [2], share fundamental molecular and cellular properties [3], [4]. Both pacemakers generate endogenous circadian rhythms of clock gene expression with periods of about 24 h, based on transcriptional/posttranscriptional negative feedback loops (TTFLs) [5], [6]. In the SCN the intracellular rhythms of TTFLs are sustained via interneuronal synchronization based upon vasoactive intestinal polypeptide (VIP) as major coupling signal [7], [8], [9], [10].

The insect neuropeptide pigment-dispersing factor (PDF) is a functional ortholog of VIP [11]–[17]. Genetic deletions suggest that PDF and VIP and their respective receptors are necessary for the expression of robust molecular and behavioral circadian rhythms in insects and mammals [7], [18]–[29]. Both, VIP- and PDF-expressing clock neurons are entrained by the light-dark cycle. In synchrony with external rhythms they couple circadian pacemaker cells to each other and gate behavioral outputs such as locomotor activity rhythms via changes of the pacemakers' electrical activity [16], [30]. Both PDF and VIP activate adenylyl cyclase (AC) via G protein-coupled receptors [31], [32]. Despite the general importance of these circadian coupling factors, their mechanisms of synchronization or gating are poorly understood [32]–[35].

A cellular mechanism of PDF-dependent gating of locomotor activity rhythms was suggested from work in the Madeira cockroach *Rhyparobia maderae* [34]. AMe neurons spike spontaneously with circadian and ultradian rhythms and form ensembles of phase-locked neurons with respect to their ultradian activity [34], [36], [37]. Application of PDF generates new ensembles of synchronized ultradian oscillators apparently via both phase-delays and phase-advances [34]. The molecular mechanisms of the effects of PDF on ultradian or circadian oscillations are not understood. Furthermore, the interdependence of ultradian and circadian rhythms in neural activity is not known. Here we show that PDF does not only signal via AC activation. PDF blocks depolarizing Na^+^- and hyperpolarizing K^+^ channels even in the same cells. Thereby it supports oscillations in intracellular Ca^2+^ levels, possibly as a prerequisite to robust synchronization.

## Materials and Methods

### Animal rearing

Madeira cockroaches (*Rhyparobia maderae*; synonym: *Leucophaea maderae*) were raised at 25°C and 50% relative humidity on 12:12 h light:dark (LD) cycles (lights on at 8:00 am) at the University of Kassel. Colonies of several hundred animals lived in plastic boxes equipped with litter and egg cartons serving as hiding places. They were fed with dry dog food, potatoes and apples at least two times per week. Water was available *ad libitum*.

### Preparation of primary cell cultures

Primary cell cultures of AMae isolated from adult males were prepared for Ca^2+^ imaging and patch clamp recordings. Between Zeitgeber time 1 and 5 hours, 4–6 AMae were isolated and incubated for 4–5 min at 37°C in 500 µl Hanks' balanced salt solution (HBSS, Gibco) containing 1 mg/ml collagenase and 4 mg/ml dispase for tissue dissociation. The enzyme solution with the dissociated cells was transferred to 10 ml Leibovitz's L-15 medium (PAA Laboratories, Cölbe, Germany) supplemented with 2.8 mg/ml yeastolate and 2.8 mg/ml lactalbumin to stop dissociations. After centrifugal sedimentation at 500 rpm for 5 min at 8°C, the cell suspension was dispersed with gentle suction and plated in 4–5 tissue culture dishes (35-mm diameter) containing 8-mm sterile glass coverslips coated with concanavalin A. The cells settled for two hours before adding 1 ml culture medium to the coverslip. The culture medium added consisted of 100 ml L-15 medium supplemented with 1 ml of 200 mg/ml glucose, 80 mg/ml fructose, 35 mg/ml L-prolin, 6 mg/ml imidazol, 1% glutamin, 0.1% gentamicin, and 2.38 mg/ml HEPES, pH 7.0, 360 mOsm/kg adjusted with NaOH and mannitol. The chemicals were obtained from Sigma-Aldrich. The culture dishes were kept in a dark humidified incubator at 20°C and were used for physiological measurements after at least 1 day and within less than a week *in vitro*. In each cell culture, 20–30 attached cells in a specified area (8×10^4^ µm^2^) were manually defined and recorded in Ca^2+^ imaging experiments. The cells were recorded before they formed long neurites. Thus, the cells were not in direct contact with each other and did not form gap junctions or synaptic interactions.

### Calcium imaging experiments

Cells were loaded in the dark with 4 µM Ca^2+^ indicator Fura-2 acetoxymethyl ester (Fura-2 AM, Molecular Probes Inc., Eugene, OR, USA) for 40 min at room temperature. Fura-2 was dissolved in DMSO and diluted in standard saline solution (in mM): 156 NaCl, 4 KCl, 1 CaCl_2_, 10 hepes, and 5 glucose (pH 7.1, 380 mOsm/kg). Following dye loading, the cover slip with dissociated cells was placed in the perfusion chamber on the stage of an Examiner D1 microscope (Zeiss, Germany) with a 20× objective (W N-Achroplan, NA 1.0). Images were acquired using Tillvision 4.0 software (Till-Photonics, Gräfelfing, Germany) with a CCD camera (Andor 885, Andor Technology Ltd, Northern Ireland). The dual wavelength Ca^2+^-sensitive indicator was excited at 340 and 380 nm with 400 ms intervals via a Polychrome 5000 monochromator (Till Photonics) with exposure times of 30 and 10 ms, respectively to calculate the ratio of fluorescence at 510 nm. As described before [38], the intracellular Ca^2+^ concentration ([Ca^2+^]_i_) was calculated according to [39]. A perfusion system with two pumps (REGLO Digital MS-2/6, Ismatec, IDEX Health&Science, Germany) connected to the recording chamber was used to alternate the flow of the normal standard saline and the stimulation solution (1 ml/min). The neurons were superfused continuously with normal standard saline. The PDF solution (PDF: NSEIINSLLGLPKVLNDA, Iris Biotech, Marktredwitz, Germany) was applied either by bath application (100 nM; 250 nM; 500 nM; 1 µM) or by pressure ejection (100 µM, 250 ms) with a Picospritzer II (General Valve Corporation, Fairfield, New Jersey, USA) via a glass pipette placed near the recorded neuron. To analyze the ion channels underlying the regular calcium transient activity, we tested the antagonists DK-AH 269 (10 µM, blocks hyperpolarization- and cyclic nucleotide-dependent (HCN) cation channels), tetrodotoxin (TTX) (100 nM, blocks Na^+^ channels), tetraethylammonium (TEA), which blocks K^+^ channels, and mibefradil (10 µM), which blocks voltage dependent low-voltage-activated (LVA) and to a lesser extent high-voltage-activated (HVA)-type Ca^2+^ channels) (Wei and Stengl, 2012). For cAMP signaling studies, the cultured cells were incubated with the adenylyl cyclase (AC) activator forskolin (10 µM) and membrane-permeable cAMP analog 8bromo-cAMP (10 µM). The combined effect of AC inhibitor SQ 22536 (20 µM) and PDF was examined. All pharmacological agents were purchased from Sigma.

### Patch clamp recordings

Whole-cell patch clamp recordings were performed with an Axopatch 200 B amplifier (Axon Instruments, Molecular Devices, Union City, CA) at room temperature and ambient light. The experiments were performed between 9:00 am and 7:00 pm within two days after preparation of the cultures. Cells were viewed with an inverted microscope (Zeiss, Axiovert) at 40× magnification.

Glass-microelectrodes with a tip resistance of 4–7 MΩ were pulled from thick-walled borosilicate glass capillaries (GC-150F-7.5, Clark Electromedical Instruments, Reading, UK) with a DMZ-Universal-Puller (Zeitz Instruments, Martinsried, Germany). The intracellular pipette solution contained 160 mM KCl, 1 mM CaCl_2_, 11 mM EGTA, 10 mM HEPES adjusted to pH 7.1 using KOH and an osmolarity of 355 mOsm/kg. The standard extracellular solution contained 156 mM NaCl, 5 mM glucose, 10 mM HEPES, 4 mM KCl, 1 mM CaCl_2_ adjusted to pH 7.1 using NaOH and an osmolarity of 380 mOsm/kg using mannitol. Seal resistances were 1–20 GΩ. Currents were digitally sampled at 50 kHz and low-pass filtered at 5 kHz with an 8-pole Bessel filter. Data acquisition and analysis were performed with pClamp 9.2 software (Axon Instruments). All AMe neurons were clamped at a resting potential of −60 mV before achieving the whole-cell configuration. Stimulations with a series of hyper- and depolarizing steps of 100 ms duration rendered current voltage curves for characterization of ion channel types. With a perfusion system PDF or ion channel blockers were added to the extracellular solution for two minutes. All current traces and current-voltage (I–V) plots analyzed for the respective experiments were recorded in the same neurons before and after treatment.

### Statistics, data analysis

#### Information theory and bias correction

Normalized frequency or normalized baseline was determined as percentage of frequency (or baseline) changes within 3 min after stimulus application. To determine whether the normalized baseline or the frequency of Ca^2+^ transients (response = *r*) reflected PDF concentrations (stimulus = *s*) more reliably, information (information = *I*; entropy = *H*) was estimated according to
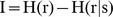
To estimate the information contained either in the normalized baseline or in the normalized frequency, we used the fact that for a given stimulus, the distribution of baseline (b) and frequency (f) could be well fitted by a Gaussian curve, after Kolmogorov–Smirnov normality test. For a given concentration, the distribution of the response could be described by
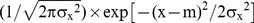
(σ_x_ = standard deviation. m = mean).

Numerical integration of the formula
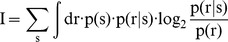
was then applied to obtain information values.

Here, *p*(*s*) = 1/4 is the probability (*p*) of the stimulus (PDF concentrations: 100 nM; 250 nM; 500 nM; 1 µM). *p*(*r*|*s*) is the probability density of the response per given stimulus *s*, and *p*(*r*) is the total probability density of the response.

Bias correction for finite sampling was performed by
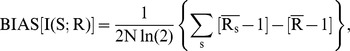
where 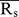 denotes the number of relevant responses (R) for the stimulus conditional response probability distribution *p*(*r*|*s*) and 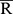 denotes the number of relevant responses for *p*(*r*).

Bias correction determined at respective system errors the number of experiments necessary to obtain reliable results.

### Comparison of calcium activity patterns of cells with regular calcium transient activity

To test whether PDF renders activity patterns with more similar phase relations than before (as expected for PDF-dependent ensemble-formation), we determined all of the time points, at which cells with regular calcium transients showed calcium peaks (T_peak_). We calculated the correlation coefficients (CCs) of the calcium levels at all T_peaks_ between any cell pairs before and after PDF (500 nM) application by using SPSS 13.0. The Pearson's CC (−1 to +1) is sensitive to a linear relationship between two variables. The CC is a measure of “correlation” between Ca^2+^ level patterns of two cells. The phases were measured with respect to the time point of PDF application. Positive values indicate that the Ca^2+^ level patterns are “in phase” and Ca^2+^ peaks of two cells occur at the same time. Negative values indicate that the activity patterns of these two cells are “out of phase”. CC_control_ and CC_PDF_ for the corresponding cell pairs were compared by paired T test (SPSS 13.0).

#### Analysis of patch clamp experiments

Responses to PDF were quantified by calculating I–V curves for the currents measured before and after PDF application (Clampfit 9.2, Molecular Devices), calculating the respective curvilinear integrals of the I–V curves, and by determining their percentage deviation (Excel 2007, Microsoft; Origin 8.6, OriginLab Corporation, Northampton, MA; Prism 5, GraphPad Software, Inc., La Jolla, CA). All data are expressed as means ± SEM.

## Results

Previous extracellular recordings from the AMe of the Madeira cockroach suggest that PDF can synchronize, activate, or inactivate pacemaker neurons [34]. To study molecular mechanisms of PDF-signaling we applied PDF to primary cell cultures of the AMe of the Madeira cockroach in calcium imaging and patch clamp experiments.

### Effects of PDF on calcium activity in primary cell cultures

**Figure 1:**
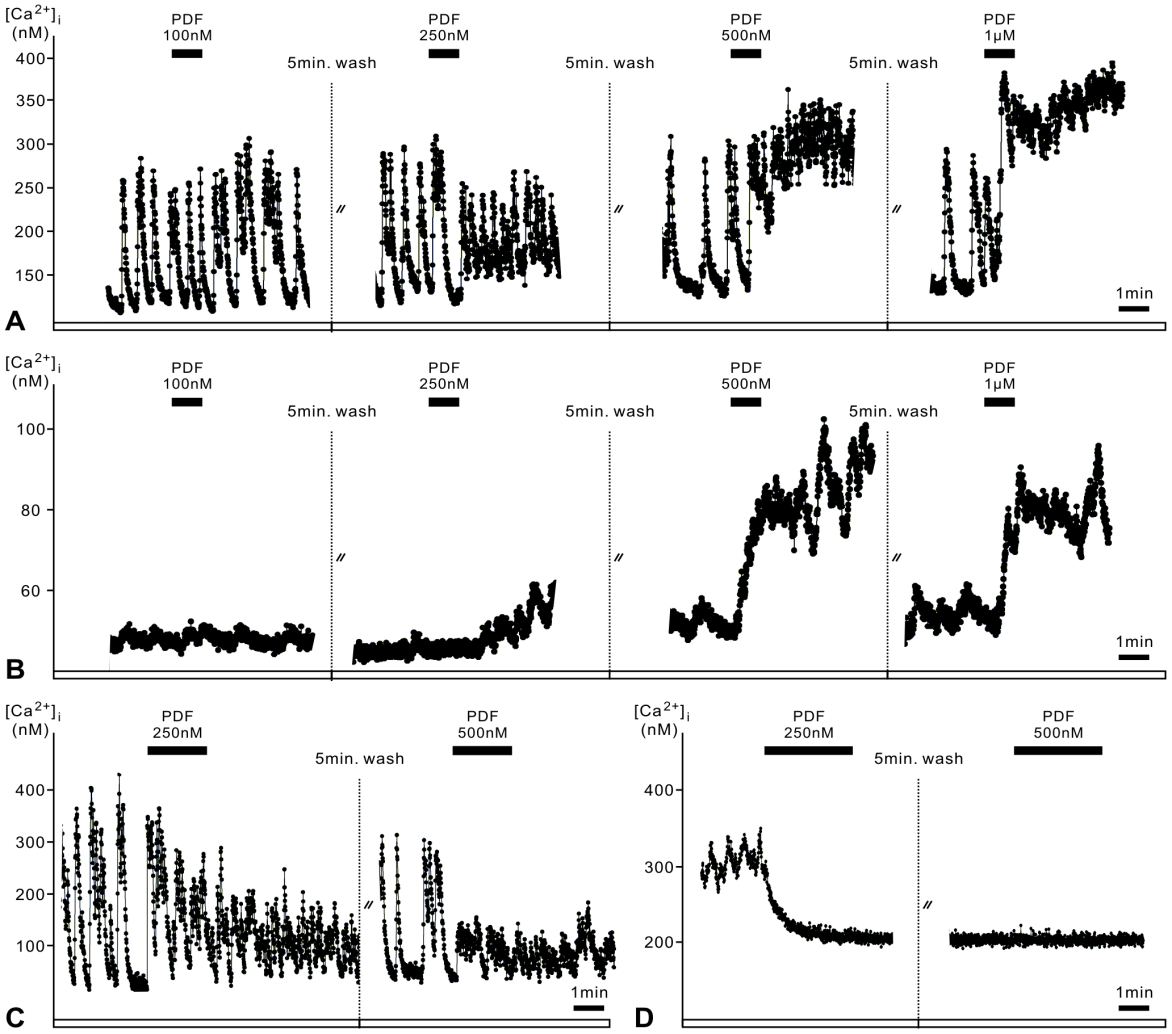
Application of PDF to accessory medulla (AMe) pacemakers in primary cell culture allows to distinguish 4 different response types 1–4 in Ca^2+^ imaging experiments. **A**. Type 1 AMe neurons express regular Ca^2+^ transients. PDF increases the frequency of spontaneous Ca^2+^ transients and the Ca^2+^ baseline dose-dependently and reversibly. **B**. Type 2 AMe neurons are not spontaneously active and have low baseline Ca^2+^ levels. PDF slowly increases Ca^2+^ baseline levels. **C**. Type 3 cells are less regularly spontaneously active than type 1 cells. PDF application slightly increases baseline Ca^2+^ levels while suppressing high amplitude Ca^2+^ transients. **D**. Type 4 AMe neurons have a high Ca^2+^ baseline level which is decreased by PDF application.

In Ca^2+^ imaging experiments (96 AMae of 63 cell cultures) bath application of PDF to dispersed AMe pacemaker neurons (n = 1526) for 1 min produced four different types of response pattern in a dose-dependent manner (**Fig. 1**).

The type 1 AMe neurons showed spontaneously occurring regular, large-amplitude Ca^2+^ transients (∼3%; 51 of 1526 AMe neurons). Bath application of PDF increased the Ca^2+^ baseline concentration and also the frequency of oscillating Ca^2+^ transients in 58.8% of type 1 AMe neurons (n = 30 of 51) recorded (**Fig. 1A**). The threshold concentration for the PDF effect varied between 100 nM and 250 nM. The PDF responses were dose-dependent and reversible. Besides regularly active type 1 pacemaker neurons, other AMe neurons were less regularly active or not active at all. The PDF-sensitive type 2 neurons were silent, non-spiking AMe cells, with low intracellular Ca^2+^ baseline levels, indicative of hyperpolarized membrane potentials. They transiently increased intracellular Ca^2+^ baseline levels after PDF application (n = 29 of 792 silent recorded cells) (**Fig. 1B**). The PDF-dependent Ca^2+^ increase occurred with different kinetics in type 2 AMe cells. The PDF-sensitive type 3 cells were not silent as type 2 cells but produced large Ca^2+^ transients which were less regular as compared to those of type 1 cells. In type 3 cells PDF increased the Ca^2+^ baseline concentration only slightly or not at all. The increase of the Ca^2+^ baseline was not dependent on the PDF concentration (**Fig. 1C**). In addition, PDF strongly decreased the amplitude of large Ca^2+^ transients in type 3 cells before blocking oscillating large Ca^2+^ transients altogether (n = 4 of 389 irregularly active AMe neurons). Type 4 cells had elevated intracellular Ca^2+^ levels which rapidly decreased following PDF application (n = 8 of 294 AMe cells with elevated intracellular Ca^2+^ baseline levels) (**Fig. 1D**).

### PDF modulates at least two counteracting currents

**Figure 2:**
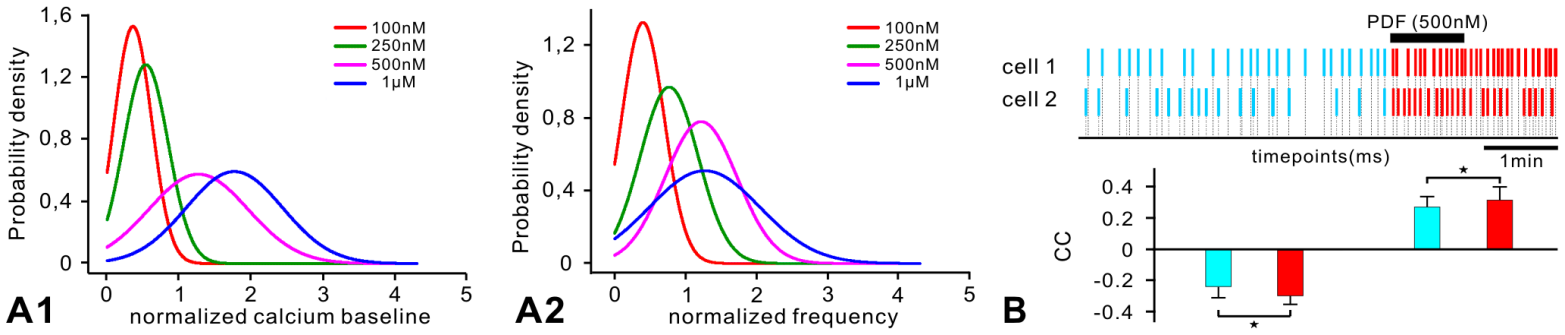
Type 1 neurons appear to be mostly depolarized and differentially synchronized by PDF. Gaussian fits for the distribution of normalized calcium baseline changes (**A1**) and normalized frequency changes (**A2**) corresponding to the 4 different PDF concentrations were obtained for type 1 cells. Only baseline changes, indicative of membrane potential changes, reliably predicted PDF concentration. **B**. Correlation coefficients of calcium levels at the timing of Ca^2+^ peaks between any two isolated, dispersed pacemakers before (blue) and after PDF (500 nM) application (red) indicate that PDF decreases the phase difference between cell pairs with “in phase” activity (direct correlation), as well as pairs with “out of phase activity” (inverse correlation) (paired t-test, with standard error of the mean, SPSS 13.0). Top panels indicate the timing of Ca^2+^ peaks in an example of two PDF-sensitive type 1 neurons.

We focused on type 1 cells, because they could be identified reliably in the primary cell cultures due to their spontaneously occurring regular, large-amplitude Ca^2+^ transients (**Fig. 1A**). In addition, since the regular Ca^2+^ transients most likely are caused by regular action potential bursts, the type 1 cells resemble an AMe cell type which was characterized previously in intracellular recordings [40], [41]. We examined whether both PDF-dependent effects (i.e., the Ca^2+^ baseline concentration rise and the increase in frequency of oscillating Ca^2+^ transients) were based on the same, or on different mechanisms. Probability density functions and information contents (see Materials and Methods) were calculated for both PDF-dependent variables (the percentage change of baseline and the frequency of oscillating Ca^2+^ transients after PDF application; **Fig. 2A_1–2_**) to test whether they encode PDF-concentration changes independently of each other. The Ca^2+^ baseline increased dose-dependently with PDF-concentration because the probability density functions at four different PDF-doses (100 nM; 250 nM; 500 nM and 1 µM) showed only partial overlap with 0.61 bits information content (**Fig. 2A_1_**). However, the PDF-dependent increase in the frequency of Ca^2+^ transients was less well correlated with PDF-concentration since probability density functions at the four concentrations overlapped more strongly and contained only 0.37 bits of information (**Fig. 2A_2_**). Thus, at the highest PDF concentration (1 µM) tested a lower frequency of Ca^2+^ transients occurred in combination with a higher baseline. Therefore, baseline-changes provided significantly more information about PDF concentrations than the frequency-changes of Ca^2+^ transients. Using Gaussian fits of distributions, the bias corrections were 0.02 bits for baseline and 0.07 bits for frequency-changes which indicated that the systems error was small enough and the sample sizes (n = 30) large enough to consolidate both PDF-effects.

To determine whether PDF was able to synchronize the ultradian activities of circadian pacemaker neurons also in isolation without synaptic connections we examined the timing of Ca^2+^ transients of all type 1 cells before and after PDF application in primary cell cultures of the AMe (n = 30) and compared all type 1 neurons pair-wise. Analysis of the timing of Ca^2+^ transients ( = the phase) before and after application of PDF (500 nM) revealed two different PDF-response groups amongst the type 1 cells which differed in phase. PDF significantly decreased the phase differences of Ca^2+^ transients within each group, while increasing the phase-difference between the two groups (**Fig. 2B**). Also, PDF always increased the frequency of Ca^2+^ transients, thereby reducing differences in frequency between cells. Thus, PDF promoted synchronization within two groups of pacemaker neurons which apparently differed in phase. PDF did not synchronize both groups with each other, but rather enhanced their differences.

### Analysis of PDF-response type 1 pacemakers in Ca^2+^ imaging experiments

**Figure 3:**
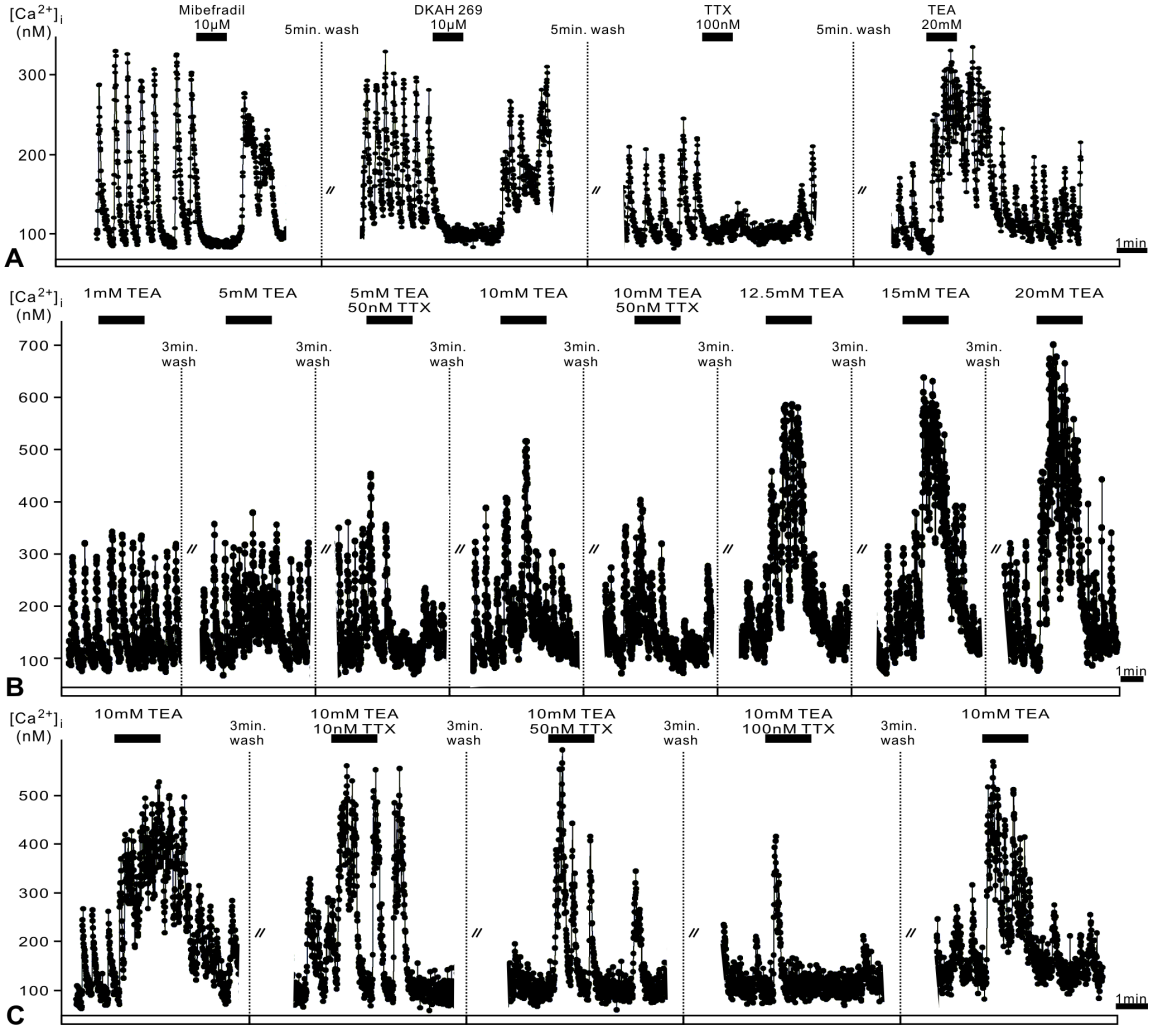
The spontaneous, regular activity of type 1 neurons depends on low voltage-activated (LVA) Ca^2+^ channels and on hyperpolarization-activated cyclic nucleotide-gated (HCN) pacemaker currents. **A**. Type 1 AMe neurons express regular Ca^2+^ transients, which can be blocked by the voltage-dependent Ca^2+^ channel antagonist mibefradil (10 µM), the HCN-channel antagonist DK-AH 269 (10 µM; which also reduces the baseline), and the Na^+^ channel antagonist TTX (100 nM). Consecutive recordings during constant perfusion of the same pacemaker neuron reveal that TEA-dependent block of K^+^ channels does not mimic all PDF effects in type 1 cells. **B**. Type 1 pacemakers increase baseline Ca^2+^ levels, amplitude, and frequency of Ca^2+^ transients with increasing concentrations (1–20 mM) of the K^+^ channel blocker TEA. Coapplication of the Na^+^ channel antagonist TTX (50 nM) increases the speed of TEA effects while truncating their durations, favoring burst-activity. **C**. Increasing the concentration of TTX (10–100 nM) finally blocks spontaneous activity and shortens TEA responses to a brief burst.

Spontaneous Ca^2+^ transients of response type 1 cells were completely abolished (n = 16) after application of the antagonist of voltage-dependent Ca^2+^ channels mibefradil (10 µM), of the reversible antagonist of hyperpolarization- and cyclic nucleotide-gated (HCN) cation channels DK-AH 269 (10 µM), or of the Na^+^ channel antagonist TTX (100 nM) (**Fig. 3A**). Thus, spontaneous Ca^2+^ transients depend on voltage-dependent Ca^2+^ and Na^+^ channels, as well as on HCN pacemaker channels. The Na^+^ channel blocker never changed the baseline level of intracellular Ca^2+^ concentrations, indicating that sustained Na^+^ channels are not responsible for the control of the resting membrane potential of type 1 neurons. In contrast, HCN-channel and Ca^2+^ channel antagonists decreased the baseline level of intracellular Ca^2+^ concentrations, at least during longer exposure times (**Figs. 3A** **,** **4D**). Mibefradil blocks low-voltage-activated (LVA)- and high-voltage-activated (HVA)-type Ca^2+^ channels, suggesting that LVA-type Ca^2+^ channels in addition to HCN-pacemaker channels contribute to spontaneous membrane potential depolarizations of type 1 pacemaker cells. These depolarizations activate in turn voltage-dependent Na^+^ channels leading to bursts of Na^+^-based action potentials. The latter initiate the upstroke of Ca^2+^ transients via activation of HVA-type Ca^2+^ channels which generate fast Ca^2+^ influx. The rapid Ca^2+^ rises then activate small conductance calcium-activated (SK) -type K^+^ channels which, together with voltage-dependent K^+^ channels, repolarize the pacemaker cells back to baseline resting potentials, as shown previously [38]. In support of this scheme, inhibition of delayed rectifier type K^+^ channels by 20 mM tetraethylammonium (TEA) increased both, the Ca^2+^ baseline and the Ca^2+^ transients (n = 18) (**Fig. 3A**). Therefore, the regular bursting activity characteristic for type 1 cells depends on HCN- and LVA-type Ca^2+^ channels as driving pacemaker channels and not on sustained opening Na^+^ channels.

**Figure 4:**
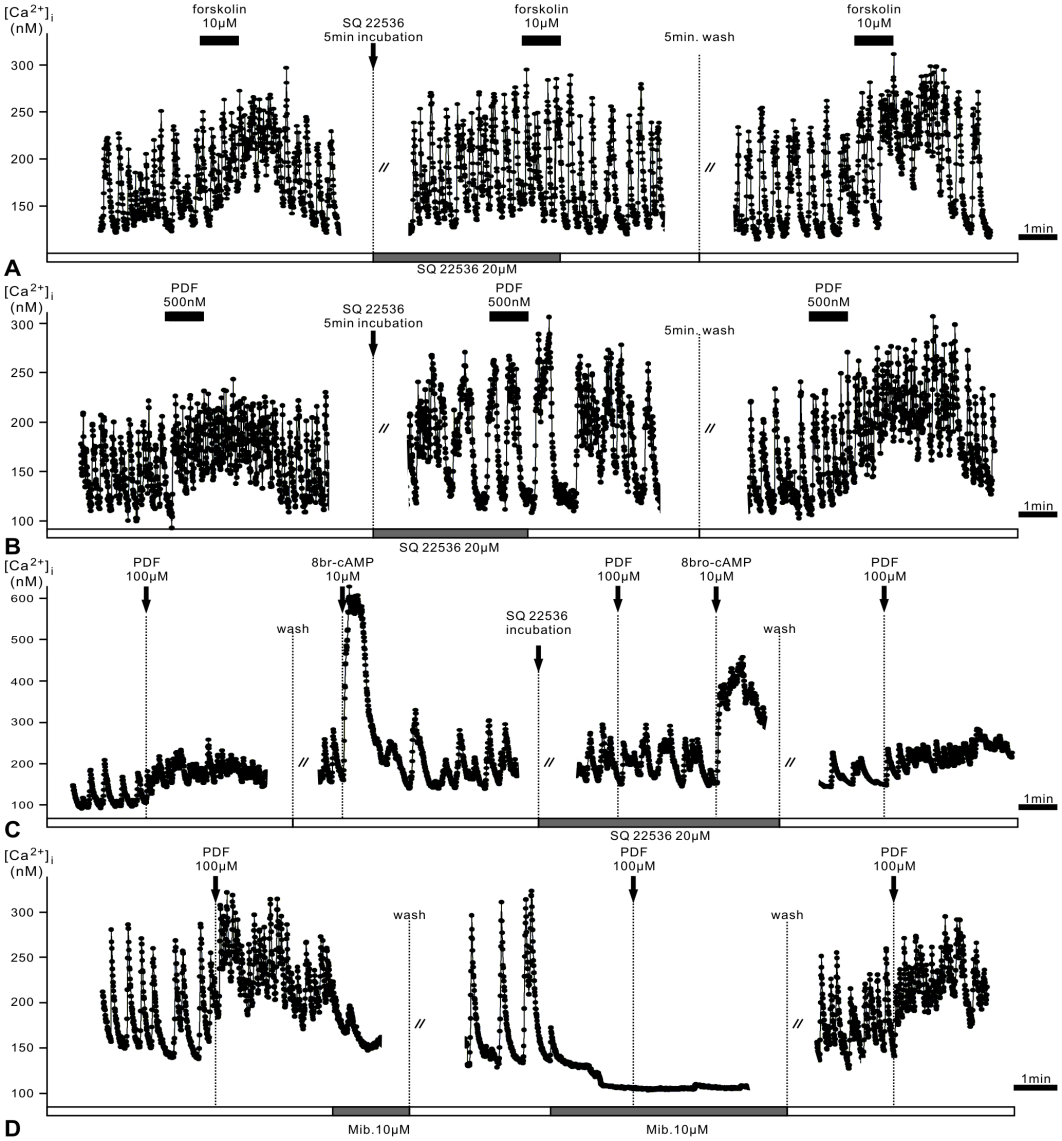
PDF responses of type 1 pacemakers are mediated by cAMP-dependent pathways. In addition, PDF effects appear to depend on intracellular Ca^2+^ baseline levels and thus, most likely on the membrane potential. **A**. Adenylyl cyclase (AC) inhibitor SQ22536 (20 µM) blocks forskolin (AC activator, 10 µM) -dependent rise of the Ca^2+^ baseline and Ca^2+^ activity in this continuous recording of type 1 AMe cells. **B**. Also, SQ22536 (20 µM) reversibly blocks the PDF-induced increase of the Ca^2+^ baseline and Ca^2+^ activity in type 1 cells. **C**. Application of 8-bromo cAMP (10 µM) produces rapid and large increases in Ca^2+^ levels, which were not blocked by SQ22536 (20 µM). **D**. Incubation of the Ca^2+^ channel antagonist mibefradil (10 µM) inhibits spontaneous activity and decreases Ca^2+^ baseline levels apparently causing membrane potential hyperpolarizations. Mibefradil-dependent effects prevent PDF (100 µM) responses in type 1 AMe neurons.

To examine whether PDF affected opposing K^+^ and Na^+^ currents (K^+^ outward currents hyperpolarize, Na^+^ inward currents depolarize) in type 1 pacemakers to different extent, we tested in co-application experiments whether a combination of TEA-dependent block of K^+^ outward currents and TTX-dependent block of Na^+^ inward currents in type 1 cells could simulate the different PDF-effects in this cell type (**Fig. 3 B,C**). Increasing concentrations of TEA transiently increased the Ca^2+^ baseline concentration as well as the amplitude and frequency of Ca^2+^ transients. Since the TEA-dependent increase in the baseline at the highest TEA concentrations was not accompanied by a decrease in amplitude and/or frequency of the Ca^2+^ transients TEA-dependent depolarizations did not result in Na^+^ channel inactivation, even if the amplitude of depolarizations was even higher than PDF-dependent depolarizations. Thus, PDF-effects in type 1 cells cannot only be due to K^+^ channel block. In contrast, co-application of TTX decreased the rise in baseline as well as the amplitude and frequency of Ca^2+^ transients. Furthermore, the TEA effects became more rapid and more transient and favored cell bursting after previous TTX addition. Thus, a combined PDF-dependent block of inward and outward currents can generate bursting. Depending on the TTX concentrations added, the TEA-dependent tonic increase in activity was truncated to a brief burst, or the spontaneous activity of type 1 cells was even deleted completely (**Fig. 3C**). Thus, differential modulation of opposing K^+^ and Na^+^ channels resulted in slow tonic or accelerated phasic excitations, -inhibitions, or only subthreshold membrane potential oscillations of the pacemaker neurons. However, the kinetics differed significantly from the slower, very long-lasting PDF-effects observed, suggesting that additional variables of PDF-signaling determine the kinetics of the PDF response.

### PDF responses in type 1 cells are mediated via adenylyl cyclase (AC) activation

Next, we examined whether PDF responses were mediated via activation of AC (**Fig. 4A–C**) causing rises of intracellular cyclic AMP concentrations, as reported for *Drosophila* [24], [42]. As positive control of cAMP signaling the AC activator forskolin (10 µM) was bath-applied (**Fig. 4A**). Forskolin increased the Ca^2+^ baseline level as well as the frequency of spontaneous Ca^2+^ transients in all type 1 cells recorded, mimicking PDF-effects (n = 5). Pre-incubation with AC inhibitor SQ22536 (20 µM) reversibly blocked the response to forskolin (**Fig. 4A**), confirming that forskolin responses resulted from activation of AC (n = 5). To determine whether the kinetic of PDF-responses was caused by slow bath application of PDF we also employed rapid PDF-application via pressure ejection by a Picospritzer. Similar to bath applications of PDF (500 nM, 1 min) (**Fig. 1A**) pressure ejection of PDF (100 µM, 250 ms) (arrows, **Fig. 4C, D** **, Fig. S1**) resulted in long-lasting PDF responses in AMe neurons. Thus, the PDF-receptor is not very sensitive to the steepness of concentration changes of its ligand. Bath application of SQ22536 (20 µM) did not block responses to the membrane-permeable cAMP analog 8-bromo cAMP (10 µM), but blocked all PDF responses of type 1 cells in bath application and pressure ejection experiments (n = 12) (**Fig. 4B,C**). Therefore, all PDF-responses of type 1 pacemakers depend on AC/cAMP signaling. In 3 of 12 cells SQ22536 (20 µM) decreased the frequency of spontaneous Ca^2+^ transients already before PDF application confirming that cAMP-dependent ion channels such as the HCN-pacemaker channel determine spontaneous Ca^2+^ activity in type 1 cells (**Fig. 4B,C**).

Addition of the voltage-dependent Ca^2+^ channel antagonist, mibefradil (10 µM), inhibited Ca^2+^ transients, decreased the baseline Ca^2+^ concentrations before PDF application, and prevented PDF responses in all type 1 cells tested (n = 4) (**Fig. 4D**). Also, block of HCN channels (**Fig. S1**) decreased baseline Ca^2+^ levels and abolished the PDF responses of type 1 cells (n = 2). Thus, in type 1 neurons PDF apparently could not increase Ca^2+^ levels in hyperpolarized cells (**Fig. 4D** **, Fig. S1**). Therefore, in type 1 cells PDF cAMP-dependently blocks at least two types of voltage-dependent ion channels, which need to be opened first via depolarization.

### PDF responses in type 2 cells are not mediated by AC/cAMP signaling

**Figure 5:**
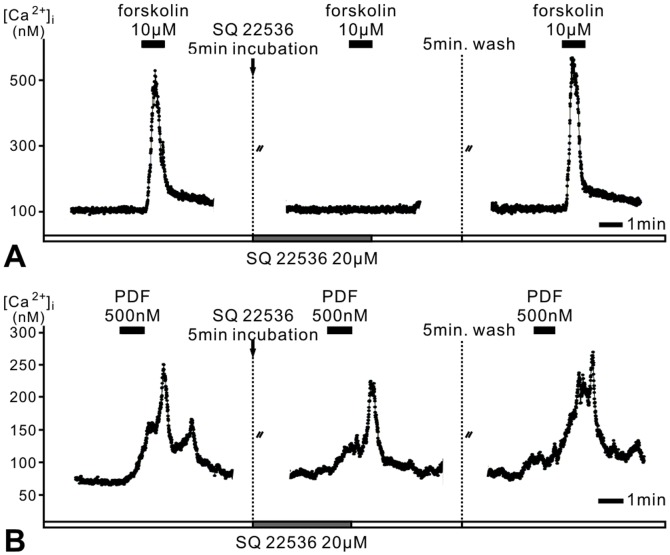
PDF response type 2 is not mediated by adenylyl cyclase (AC) activity. **A**. Preincubation with the AC inhibitor SQ22536 (20 µM) blocks the forskolin (10 µM) -induced increase of Ca^2+^ in silent AMe cells. **B**. In contrast, SQ22536 does not affect PDF responses in type 2 neurons.

Like in type 1 cells bath application of forskolin (10 µM) increased the intracellular Ca^2+^ concentration also in silent type 2 AMe cells (n = 9). The forskolin response was blocked reversibly by SQ22536 (20 µM) **(** **Fig. 5A**). However, preincubation of SQ22536 (20 µM) never inhibited the PDF response in type 2 pacemaker neurons (n = 9). Therefore, PDF responses of type 2 pacemakers are not mediated by AC/cAMP signaling (**Fig. 5B**). The slow kinetics of the PDF-dependent strong, transient increase in the baseline Ca^2+^ concentration of type 2 neurons hinted that PDF did not directly activate Ca^2+^ channels. Furthermore, PDF responses did not resemble a block of voltage-dependent K^+^ channels, which would result in rapid depolarization-dependent opening of voltage-dependent Ca^2+^ channels (**Fig. 3B**).

### Application of PDF inhibits outward K^+^ and inward Na^+^ currents in AMe neurons

**Figure 6:**
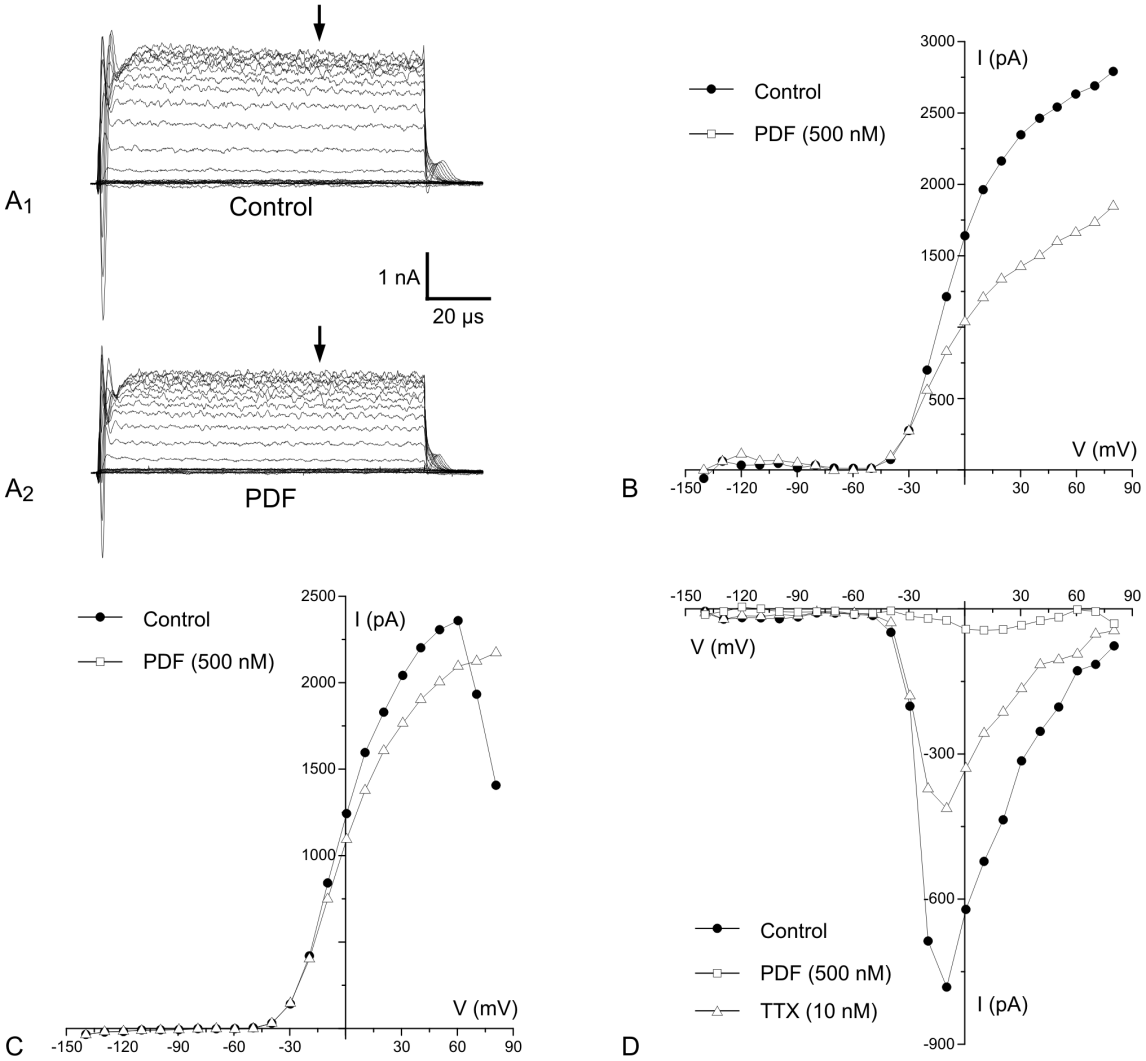
Application of PDF blocks outward K^+^ and inward Na^+^ current components. In whole-cell patch clamp recordings AMe neurons in primary cell cultures were kept at a holding potential of −60 mV. Voltage-dependent currents were activated by series of depolarizing voltage steps from −140 mV to +80 mV with 10 mV increments. **A_1_–A_2_**. Current traces before and after application of 500 nM PDF (2 min) to the extracellular solution indicate that PDF inhibits part of a delayed rectifier type potassium (K^+^) outward current. **B**. The I-V plot of the same recording at the position indicated by the arrows in **A_1_ and A_2_** shows the decline of outward currents while sustained, small inward currents, which counteract outward K^+^ currents at hyperpolarized potentials, are not affected. **C**. In another recording PDF blocks Ca^2+^-dependent outward K^+^ currents, which cause the characteristic downward bend of the outward currents, while apparently not affecting delayed rectifier type K^+^ currents, or small sustained inward Ca^2+^ currents which counteract K^+^ outward currents at hyperpolarizing potentials. **D**. In another AMe neuron PDF inhibits voltage-gated fast Na^+^ inward currents that activate around −40 mV. Washing in of PDF-free saline containing the Na^+^ channel antagonist tetrodotoxin (TTX) almost competely blocks the residual inward current.

To examine which ion channels were affected by PDF application whole-cell patch clamp experiments were performed on primary cell cultures of adult AMe neurons (n = 83). Because we could not identify type 1–4 cells previously characterized in Ca^2+^ imaging experiments according to their morphology or their current responses, we did not select for a specific morphological cell type. Instead, we concentrated on the analysis of delayed rectifier type K^+^ outward and Na^+^ inward currents as the most likely PDF-targets (**Fig. 6A–D**). The analysis of PDF-effects on non-specific cation currents such as the HCN pacemaker current, of Cl^−^-, and of Ca^2+^ currents was left aside for future studies.

Immediately after reaching the whole-cell configuration, membrane potentials *V_m_* = −41±5 mV (n = 83) were measured in the current-clamp mode, indicating that AMe pacemaker neurons lie close to spike threshold. To investigate the effects of PDF, voltage steps from −140 mV to +80 mV in 10 mV increments were applied and current voltage (I–V) curves were obtained before and after bath-application of 500 nM PDF (n = 32). As determined by their typical I–V relations or by pharmacological experiments, all pacemaker neurons examined expressed prior to PDF application delayed rectifier-type outward K^+^ currents (n = 32), while only 10 of 32 cells showed Ca^2+^-activated K^+^ outward currents and 12 of 32 fast, voltage-gated, TTX-sensitive Na^+^ inward currents. About 38% of all AMe neurons tested (12 of 32) responded to PDF application. In 10 PDF-sensitive pacemaker neurons PDF inhibited delayed rectifier type outward currents (**Fig. 6A_1_, A_2_, B**). In these cells PDF blocked 37±4% of the total delayed rectifier outward currents. In 40% of neurons with Ca^2+^-dependent K^+^ currents (4 out of 10) PDF reduced the Ca^2+^ –dependent K^+^ currents **(** **Fig. 6C**). In 3 of the 4 cells apparently also delayed rectifier type K^+^ currents, but not Na^+^ inward currents were reduced via PDF. We found no evidence for PDF-dependent modulation of I_A_-type K^+^ channels in the neurons tested. Voltage-gated, TTX-sensitive, fast, transient inward currents activating at about −40 mV were blocked by PDF in 25% of the AMe neurons with Na^+^ currents (3 out of 12) (**Fig. 6D**). In these cells PDF inhibited 31%±7% of the respective inward current. In 2 of the 3 cells, showing a PDF-induced reduction of Na^+^ currents, the delayed rectifier-type, but not the Ca^2+^-activated K^+^ channels were also blocked by PDF.

## Discussion

**Figure 7:**
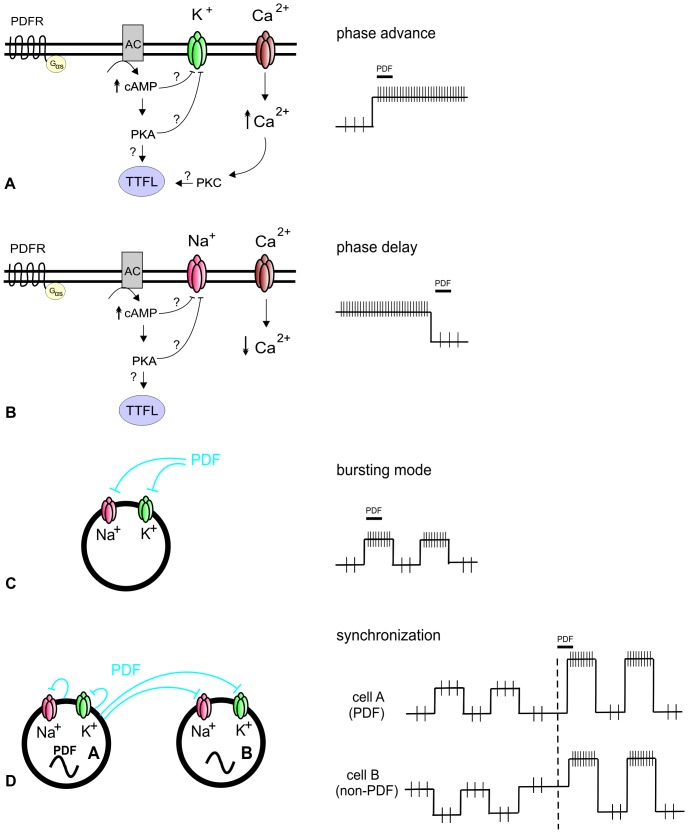
Hypothetical model of PDF signaling in spontaneously active type 1 circadian pacemaker neurons. **A**. We suggest that PDF signals via adenylyl cylcase activity in type 1 cells. The cAMP-dependent block of K^+^ channels depolarizes the cell and thereby opens voltage-gated Ca^2+^ channels. The resulting rise in intracellular Ca^2+^ together with the rise in cAMP concentrations then feeds back to the molecular clockwork (TTFL) via activation of PKA and PKC, thereby phase-advancing circadian rhythms of circadian pacemaker neurons. **B**. In contrast, PDF-dependent block of Na^+^ channels hyperpolarizes and thereby phase-delays pacemaker neurons only via PKA, but not concomitant PKC-dependent feedback to the TTFL. **C**. When PDF blocks both K^+^ and Na^+^ channels it promotes rhythmic membrane potential oscillations and causes bursting. **D**. Finally, PDF promotes fast synchronization between two pacemakers, which are coupled via their common PDF-sensitivity. If the PDF releasing pacemaker is also PDF-sensitive, because of autoreceptor expression [48] it will synchronize with the postsynaptic PDF-sensitive pacemaker neuron. PDF-dependent rhythmic bursting is suggested to promote fast synchronization.

Interneuronal synchronization, which occurs at ultradian timescales, is a prerequisite to strong cellular and network level circadian rhythms [43]. Furthermore, rhythmic changes of pacemaker cell membrane potentials sustain circadian rhythms and are crucial clock components [44]–[51]. The neuropeptide PDF, a functional ortholog of the mammalian peptide VIP, is the circadian coupling signal of flies and cockroaches necessary for synchronized circadian clock gene expression and synchronized locomotor activity rhythms [30], [32]. To further elucidate the signaling mechanisms of PDF, we studied its action in Ca^2+^ imaging and patch clamp experiments on primary cell cultures of the AMe, the circadian pacemaker of the Madeira cockroach. Application of PDF increased baseline Ca^2+^ levels and changed the frequency of Ca^2+^ transients in type 1 pacemaker neurons, apparently primarily via cAMP-dependent reduction of outward K^+^ currents but also via cAMP-dependent reduction of inward Na^+^ currents. As judged by the modulation of the Ca^2+^ baseline or the frequency and amplitude of Ca^2+^ transients PDF could either depolarize (types 1,2,3 cells) and/or hyperpolarize (types 1,3,4 cells) (**Fig. 7A,B**). PDF favored bursting (type 1) (**Fig. 7 C**), switched a cell from bursting to tonic activity (type 3), or elicited transient bursts (type 2). In addition to activation of adenylyl cyclase in type 1 neurons, PDF signals via adenylyl cyclase-independent pathways in type 2 pacemakers. Furthermore, PDF could elicit oscillations (types 1,3 cells) as previously observed in extracellular recordings. We propose that PDF-dependent ultradian oscillatory activity in PDF-sensitive “pre- and postsynaptic” pacemakers is a prerequisite to their robust mutual synchronization (**Fig. 7D**).

### Type 1 pacemakers are candidates for regularly bursting bright- and dark-rhythm neurons

In our study of PDF-signaling in the circadian system we focused on type 1 pacemakers, which generated regular, large-amplitude Ca^2+^ transients. Type 1 cells are reminiscent of two types of regularly bursting AMe neurons identified previously in intracellular recordings which occured at a similar low frequency as type 1 cells [40]. Illumination of the ipsilateral compound eye activated one type (bright-rhythm cell) and inhibited the other (dark-rhythm cell) independent of light intensity. Both types generated regular membrane potential oscillations causing regular bursts of action potentials of around 40 Hz. They never responded to motion or polarized light. These AMe pacemakers had ramifications in the ipsilateral AMe and medulla and projected to the contralateral optic lobes, sending processes to the ventral protocerebrum [40]. We hypothesize that these regularly bursting bright- and dark-rhythm neurons correspond to the two groups of regularly bursting type 1 pacemakers which were synchronized differentially by PDF thereby maintaining stable phase-differences (**Fig. 2B**). These bright- and dark-rhythm neurons are ideally suited to allow for adaptation to different photoperiods via differential synchronization with PDF-sensitive pacemaker neurons controlling locomotor activity either at the beginning (evening cells = E oscillators) or the end of the night (morning cells = M oscillators) [30].

### PDF signals via adenylyl cyclase-dependent and -independent pathways

The PDF receptor (PdfR) of the fruit fly *D. melanogaster* resembles the VIP receptor VPAC-2 [31], [52]–[54]. Both are class II G protein coupled receptors that activate adenylyl cyclases [24]–[26], [31]. However, whereas VIP requires both adenylyl cyclase and phospholipase Cβ (PLCβ) to relay phase information [31], in *Drosophila* only PdfR-dependent rises in intracellular cAMP but not in Ca^2+^ concentrations were observed *in situ* [42], [55]–[57]. In the cockroach the activated PdfR leads to increases in intracellular Ca^2+^ concentrations via activation of adenylyl cyclase in type 1- and via an adenylyl cyclase-independent pathway in type 2 pacemakers. We assume that PDF in type 2 cells induces Ca^2+^ release from intracellular stores via PLCβ-stimulation, as slow kinetics suggest. Also in *Drosophila* Agrawal et al. [58] implied PdfR-dependent G_q_ signaling in flight control circuits. Thus, coupling of the PdfR to different G proteins might also occur in the fruit fly [58]. Finally, increasing evidence suggests that PDF is a systemic hormonal coupling signal which integrates multimodal sensory inputs with the internal physiological state of the insect via unknown mechanisms [20], [58], [59], [60]. Whether the long-lasting PDF responses play a role for temporal integration of multimodal inputs and whether they employ mechanisms suggested for the long-lasting VIP responses remains to be examined [35].

### PDF modulates inward and outward currents in different PDF response types

PDF application to type 1 cells increased the baseline more reliably than the frequency of action potential activity. In addition, TEA-dependent depolarizations alone could not mimic all PDF-effects. Therefore, PDF must target at least two different ion channels in type 1 cells. Furthermore, because blocker of adenylyl cyclase activity prevented PDF effects in type 1 cells PDF signals via rises of cAMP concentrations. Apparently, PDF first blocked delayed rectifier K^+^ channels in a cAMP-dependent manner, before blocking Na^+^ channels. The different time course of the PDF-effects suggest that cAMP directly affected K^+^ channels such as the eag-family of K^+^ channels [61]. Also in the fruit fly a direct, cAMP mediated PDF-effect on ion channels was suggested [62]. Then, at higher PDF concentrations, also Na^+^ channels were inactivated in cockroach pacemakers, possibly indirectly via protein kinase A (PKA)-dependent mechanism [63]. From applications of different concentrations of K^+^ which also increased the Ca^2+^ baseline we calculated that 250 nM PDF depolarizes pacemaker neurons by about 16 mV. Thus, a depolarization of 16 mV would elicit a sustained increase in the action potential frequency, but would not cause inactivation of Na^+^ channels because the majority of cAMP-insensitive K^+^ outward currents remain active. Therefore, it is most likely that in type 1 cells PDF inactivated both delayed rectifier K^+^- and Na^+^ channels in the same cells. PDF-dependent inactivation of either K^+^ and/or Na^+^ channels to varying extents could account for all other PDF-response types, except for response type 2. Furthermore, the previously observed PDF-dependent block of spiking could be explained via PDF-dependent block of Na^+^ channels, while the PDF-dependent activation could result from K^+^ channel-inactivation [34]. Which K^+^-channel types were affected and whether cation- and Ca^2+^ channels also contributed to different PDF responses remains to be examined. In mammals likewise, VIP affects different K^+^- and Na^+^ channels, but does not appear to modulate HCN pacemaker channels [33], [64]–[67]. The functional consequences of PDF's and VIP's effects on opposing ion channels for the ultradian and circadian activity of pacemaker neurons are not resolved yet, but they might promote synchronization since they promote membrane potential oscillations (**Fig. 7D**).

### How do ultradian action potential rhythms affect circadian rhythms?

In *Drosophila* PDF affects circadian rhythms of clock gene expression in the different groups of circadian pacemaker neurons, the M-oscillators and the E-oscillators which control circadian locomotor activity rhythms differently [30]. PDF delays E-pacemakers, which control the evening peak of locomotor activity, and synchronizes and advances the M-cells, which control the morning peak of locomotor activity rhythms, reminiscent of long-day conditions [30], [46], [48], [68], [69]. In the Madeira cockroach PDF injections shift circadian locomotor activity rhythms generating a phase-response curve with a prominent delay at the late day/early night and a very small, narrow advance portion in the late night/early day, quite similar to VIP [31], [70]. Furthermore, PDF inhibited, activated, or synchronized ultradian action potential rhythms of circadian pacemaker neurons [34]. It is poorly understood how changes of electrical activity at ultradian timescale relate to modulation of circadian rhythms. Nevertheless, it was suggested that VIP-dependent phase-advances of the TTFL are caused via brief strong activation of period-gene expression requiring both adenylyl cyclase and PLCβ modulation based upon rapid increases in the cell's electrical activity [31], [51]. Also in *Drosophila* changes in the membrane potential of circadian pacemaker neurons are associated with phase-shifts of clock gene-and locomotor activity rhythms [62]. Furthermore, Seluzicki et al. [71] provided evidence for PDF-dependent stabilization of TIMELESS mediated via a protein kinase A (PKA)-dependent mechanism and Li et al. [72] for PDF-dependent stabilization of PERIOD also via cAMP and PKA-dependent mechanisms which might reset and synchronize the TTFL. Therefore, it is intriguing to hypothesize that also in the Madeira cockroach a PDF-dependent strong depolarization which is accompanied by increases in intracellular cAMP and Ca^2+^ concentrations, possibly activating PKA and protein kinase C (PKC), causes phase-advances of circadian rhythms (**Fig. 7A**). In contrast, PDF-dependent hyperpolarization caused by rises in cAMP and accompanied by decreases in intracellular Ca^2+^ levels might phase-delay circadian rhythms of TTFL only by activation of PKA but not PKC (**Fig. 7B**). Finally, we suggest that PDF–dependent synchronization between two PDF-sensitive pacemakers is achieved via PDF-dependent modulation of opposing K^+^- and Na^+^-channels in the same pacemaker neuron generating ultradian membrane potential oscillations (**Fig. 7D**). Then, PDF-dependent interaction between these ultradian oscillators would cause synchronization via resonance [34], especially if the PDF-releasing “presynaptic” neuron expresses autoreceptors as reported for *Drosophila* [27], [29], [42], [48], [73], [74].

## Supporting Information

Figure S1 **Block of HCN channel with antagonist DKAH269 (10 µM) decreased the Ca^2+^ baseline and abolishes the PDF responses of type 1 cells.**
(TIF)

Table S1 **The responses of calcium baseline and frequency changes of type 1 cells corresponding to the 4 different PDF concentrations (100 nM; 250 nM; 500 nM; 1 µM).** Normalized frequency or normalized baseline was determined as percentage of frequency (or baseline) changes within 3 min after stimulus application. Gaussian fits for the distribution of normalized frequency or normalized baseline were shown.
(XLSX)

Table S2 **Correlation coefficients of calcium levels at the timing of calcium peaks between any two isolated, dispersed pacemakers before and after PDF (500 nM) application.**
(XLSX)

Table S3 **PDF-dependent reduction of I_Na_.** In whole-cell patch clamp recordings AMe neurons were stimulated with depolarizing voltage steps before and after application of PDF (2 min). I-V relationships for I_Na_ were generated and the respective curvilinear integrals (areas under the I-V curves; AUC) were used to calculate the percentage reduction of this current component.
(XLSX)

Table S4 **PDF-dependent reduction of I_K_.** In whole-cell patch clamp recordings AMe neurons were stimulated with depolarizing voltage steps before and after application of PDF (2 min). I-V relationships for I_K_ were generated and the respective curvilinear integrals (areas under the I-V curves; AUC) were used to calculate the percentage reduction of this current component.
(XLSX)
