# Supplementary figures and images for: Signaling of Pigment-Dispersing Factor (PDF) in the Madeira Cockroach Rhyparobia maderae
Source: PLoS One. 2014 Sep 30;9(9):e108757. doi: 10.1371/journal.pone.0108757 (PMC4182629; doi:10.1371/journal.pone.0108757)

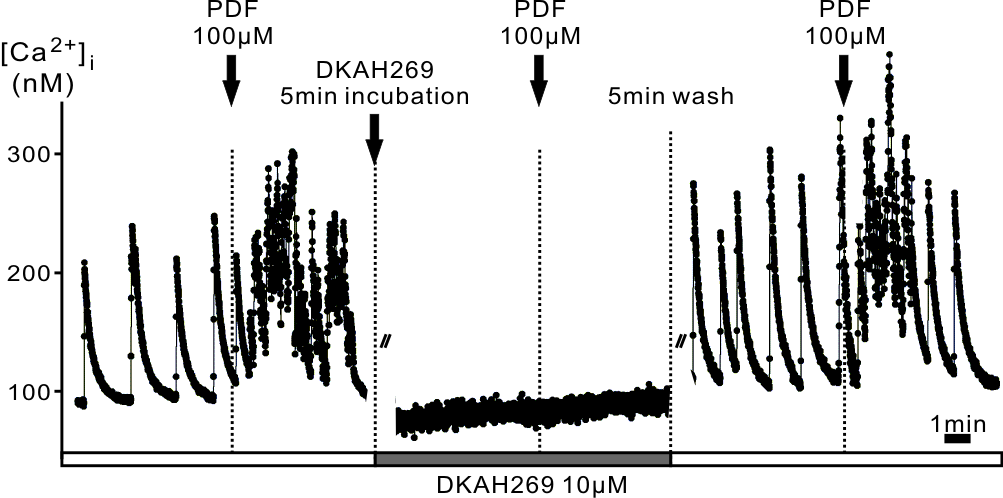

Supplement: Figure S1 — Block of HCN channel with antagonist DKAH269 (10 µM) decreased the Ca2+ baseline and abolishes the PDF responses of type 1 cells. (TIF) [file pone.0108757.s001.tif]
